# Epigenetic modification of PKMζ rescues aging-related cognitive impairment

**DOI:** 10.1038/srep22096

**Published:** 2016-03-01

**Authors:** Chen Chen, Shi-Qiu Meng, Yan-Xue Xue, Ying Han, Cheng-Yu Sun, Jia-Hui Deng, Na Chen, Yan-Ping Bao, Fei-Long Zhang, Lin-Lin Cao, Wei-Guo Zhu, Jie Shi, Wei-Hong Song, Lin Lu

**Affiliations:** 1 Institute of Mental Health, Peking University Sixth Hospital, and Key Laboratory of Mental Health, Beijing 100191, China; 2 National Institute on Drug Dependence and Beijing Key Laboratory of Drug Dependence Research, Peking University, Beijing 100191, China; 3 Department of Biochemistry and Molecular Biology, Peking University Health Science Center, Beijing 100191, China; 4 Peking-Tsinghua Center for Life Sciences and PKU-IDG/McGovern Institute for Brain Research, Peking University, Beijing 100871, China; 5 Brain Research Centre, Departments of Medicine and Psychiatry, University of British Columbia, Vancouver, British Columbia, V6T 1Z3, Canada

## Abstract

Cognition is impacted by aging. However, the mechanisms that underlie aging-associated cognitive impairment are unclear. Here we showed that cognitive decline in aged rats was associated with changes in DNA methylation of protein kinase Mζ (PKMζ) in the prelimbic cortex (PrL). PKMζ is a crucial molecule involved in the maintenance of long-term memory. Using different behavioral models, we confirmed that aged rats exhibited cognitive impairment in memory retention test 24 h after training, and overexpression of PKMζ in the PrL rescued cognitive impairment in aged rats. After fear conditioning, the protein levels of PKMζ and the membrane expression of GluR2 increased in the PrL in young and adult rats but not in aged rats, and the levels of methylated *PKMζ* DNA in the PrL decreased in all age groups, whereas the levels of unmethylated *PKMζ* DNA increased only in young and adult rats. We also found that environmentally enriched housing reversed the hypermethylation of *PKMζ* and restored cognitive performance in aged rats. Inactivation of PKMζ prevented the potentiating effects of environmental enrichment on memory retention in aged rats. These results indicated that PKMζ might be a potential target for the treatment of aging-related cognitive impairment, suggesting a potential therapeutic avenue.

Cognitive functions, especially those that involve the medial prefrontal cortex (mPFC), decline with age, and the most notable manifestation is impaired memory^1^,^2^,^3^. Conditioned fear memory, Morris water maze (MWM) and novel object recognition (NOR) are classical models of conditioned learning, spatial memory and recognition memory respectively, and are widely used to examine aging-related cognitive decline^4^,^5^,^6^,^7^.

Prefrontal synaptic plasticity plays a critical role in memory retention in aged animals. For example, the aging-related dysregulation of γ-aminobutyric acid-ergic signaling in mPFC plays a causal role in impaired working memory and spatial alternation^8^,^9^. Serotoninergic receptor antagonist reversed aging-induced deficits in MWM and NOR^10^. Reduced expression of neural cell adhesion molecule in the hippocampus and mPFC might be a critical factor for aging-related cognitive impairments in MWM and T-maze^11^. However, the molecular basis underlying aging-related cognitive decline is still poorly understood.

Epigenetic modification of chromatin, including DNA methylation, regulates the transcription of several genes that are responsible for memory formation and maintenance^12^,^13^,^14^. Aberrant changes in methylation of the Arc gene contribute to aging-related decreases in Arc transcription within the Cornu Ammonis 1 (CA1) area and dentate gyrus of the hippocampus, leading to impairment in spatial memory^15^. Dysregulation of DNA methylation in the promoter regions of three aging-relevant genes (Gabra5, Hspa5 and Syn1) in the CA3 area is correlated with poor spatial memory performance^16^. Furthermore, elevations of Dnmt3a2 levels in the hippocampus restore cognitive function in aged mice^17^. However, the specific genes whose methylation is responsible for aging-related cognitive impairment have not yet been identified.

Protein kinase Mζ (PKMζ) is synthesized from an independent mRNA that encodes the catalytic domain of protein kinase Cζ (PKCζ) without a regulatory domain^18^. PKMζ is known as a persistently active isoform of protein kinase C (PKC). In the past few years, PKMζ in multiple brain areas, such as the hippocampus^19^, mPFC^20^,^21^, amygdala^22^ and neocortex^23^, has been identified as a crucial molecule in the maintenance of long-term spatial^24^, instrumental^25^ and emotional memories^23^.

Thus, we explored whether the aberrant methylation of *PKMζ* DNA in the prelimbic cortex (PrL) is involved in aging-related memory decline and whether cognitive impairment in aged rats can be rescued through regulation of PKMζ.

## Results

### Overexpression of PKMζ in the PrL rescued the impairment in memory retention in aged rats

**Figure 1:**
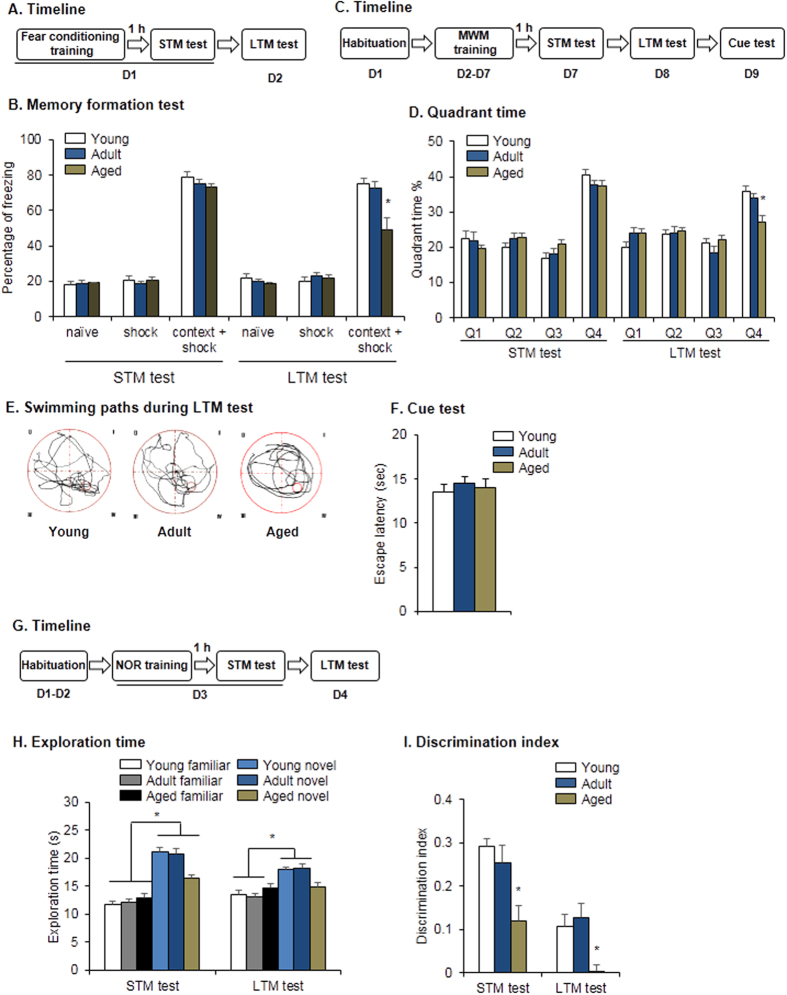
Aged rats exhibited impaired retention of LTM. (**A,B**) Aged rats exhibited impaired memory retention of fear conditioning. **(A)** Experimental timeline for fear conditioning. (**B**) Freezing scores during the STM and LTM tests. *p < 0.05, compared with young group. *n* = 6–8 per experimental condition. (**C–F**) Aged rats exhibited impaired memory retention in the MWM task. **(C)** Experimental timeline for the MWM task. (**D**) Time in each quadrant during the STM and LTM probe tests. *p < 0.05, compared with young group. *n* = 10 per experimental condition. (**E**) Swimming paths during LTM probe test. (**F**) Cue test. (**G–I**) Aged rats exhibited impaired memory retention in the NOR task. (**G**) Experimental timeline for the NOR task. (**H**) Exploration time during the STM and LTM tests. ^#^*p* < 0.05, compared with age-matched familiar group. *n* = 9–10 per experimental condition. (**I**) Discrimination index. *p < 0.05, compared with manipulation-matched young group. *n* = 9–10 per experimental condition.

We first assessed the causal relationship between decreases in the levels of PKMζ and aging-related cognitive impairment using contextual fear conditioning, the Morris water maze (MWM), and the novel object recognition (NOR) test^4^,^5^,^7^. Young rats (3 months old), adult rats (9 months old), and aged rats (24 months old) were subjected to contextual fear conditioning or three electric only as a control. All of the rats then underwent freezing tests to evaluate short-term memory (STM) and long-term memory (LTM) 1 and 24 h later (Fig. 1A). We found that aged rats exhibited normal STM (two-way analysis of variance [ANOVA], main effect of condition, *F*_2,49_ = 434.04, *p* < 0.001) but displayed decreased freezing time in LTM (two-way ANOVA, condition × age interaction, *F*_4,49_ = 3.72, *p* < 0.05; main effect of condition, *F*_2,49_ = 109.87, *p* < 0.05; main effect of age, *F*_2,49_ = 4.01, *p* < 0.05; Fig. 1B). In the MWM and NOR test, we also found that LTM was impaired in aged rats (Fig. 1C–I). In the MWM task (Fig. 1C), aged rats exhibited normal STM, whereas in the LTM test, they spent less time in the target quadrant (two-way ANOVA, main effect of quadrant, *F*_3,108_ = 40.084, *p* < 0.001; test × quadrant interaction, *F*_3,108_ = 9.415, *p* < 0.001; Fig. 1D) compared with young rats. The swimming paths revealed that young and adult rats had higher preference for the target quadrant compared with aged rats during the LTM test (Fig. 1E). All of the rats spent similar time reaching the visible platform during the cue test (*p* > 0.05; Fig. 1F). In the NOR task (Fig. 1G), although all rats exhibited significant preference for the novel object during the STM test (Fig. 1H), aged rats had a much lower discrimination index (one-way ANOVA, *F*_2,26_ = 3.365, *p* = 0.049; Fig. 1I). During the LTM test, young and adult rats had significant preference for the novel object (two-way ANOVA, main effect of object, *F*_*1,52*_ = 12.087, *p* = 0.001; Fig. 1H), whereas aged rats exhibited no preference between the familiar and novel objects and thus had a much lower discrimination index (one-way ANOVA, *F*_2,26_ = 3.365, *p* = 0.049; Fig. 1I).

**Figure 2:**
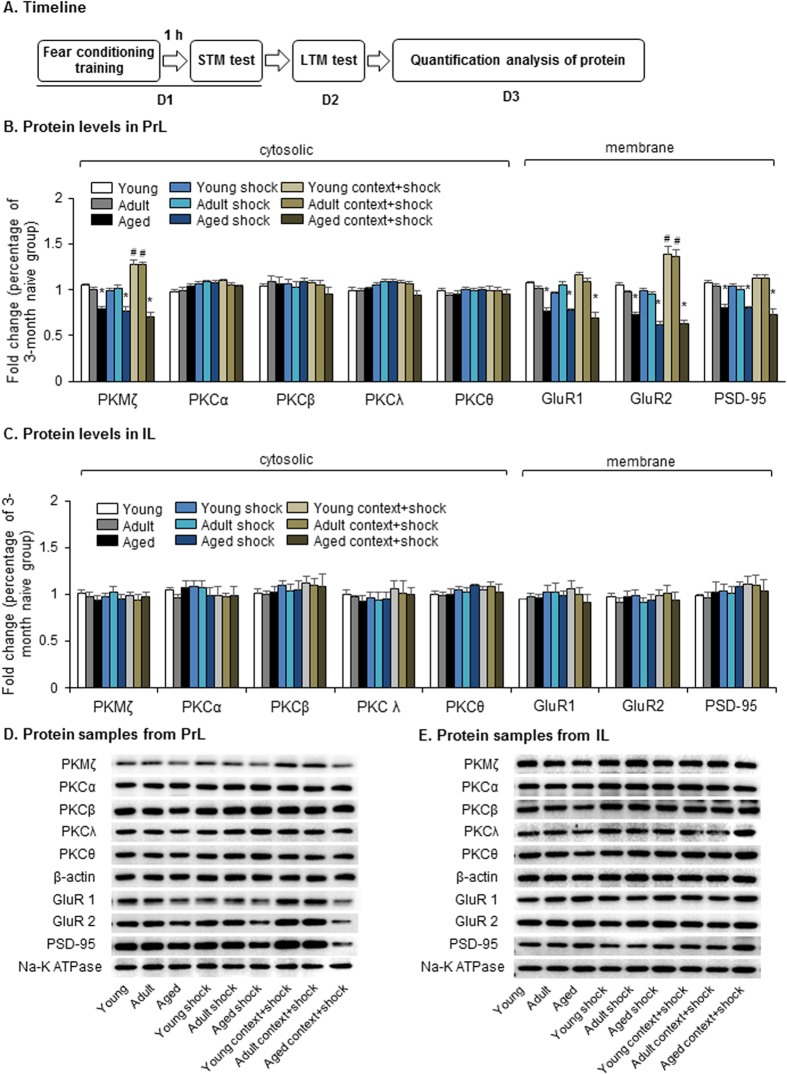
Aged rats exhibited decreased PKMζ levels in the PrL. (**A**) Experimental timeline for detection of cytosolic and membrane protein levels. Cytosolic and membrane protein levels and representative Western blots in the PrL (**B**,**D**) and IL (**C**,**E**) after the LTM test. Levels of membrane proteins including GluR1, GluR2 and PSD-95 were normalized to Na, K-ATPase and levels of cytosolic proteins like PKMζ, PKCα, PKCβ, PKCλ, and PKCθ were normalized to β-actin. ^#^*p* < 0.05, compared with age-matched naive group; *p < 0.05, compared with condition-matched young group. *n* = 6 per experimental condition.

We then assessed the cytosolic expression of PKMζ and other isoforms of PKC, including PKCα, PKCβ, PKCλ, and PKCθ, in the PrL and infralimbic cortex (IL) in rats at different ages after the LTM test (Fig. 2A). We found that contextual fear conditioning increased the cytosolic levels of PKMζ in the PrL in young and adult rats, but no such effect was found in shock groups. Significant decreases in PKMζ were observed in the naïve, shock and conditioned training groups of aged rats (two-way ANOVA, main effect of condition, *F*_2,45_ = 12.004, *p* < 0.001; main effect of age, *F*_2,45_ = 59.571, *p* < 0.001; condition × age interaction, *F*_4,45_ = 6.336, *p* < 0.001; Fig. 2B,D). No difference was found in the expression of cytosolic PKCα, PKCβ, PKCλ, or PKCθ (all *p* > 0.05). Previous research showed that PKMζ regulates the activity of glutamate receptor 2 (GluR2)-containing AMPA receptors and the expression of postsynaptic density-95 (PSD-95) protein^22^,^26^. Thus, we also examined the membrane expression of GluR1, GluR2, and PSD-95. We found that baseline levels of all three proteins decreased in aged rats, and fear conditioning increased the membrane levels of GluR2 in the PrL in young and adult rats but did not increase the expression of GluR1, GluR2 or PSD-95 in the PrL in aged rats (two-way ANOVA, main effect of age for GluR1, *F*_2,45_ = 46.505, *p* < 0.001; condition × age interaction for GluR1, *F*_4,45_ = 2.804, *p* < 0.05; main effect of condition for GluR2, *F*_2,45_ = 14.812, *p* < 0.001; main effect of age for GluR2, *F*_2,45_ = 51.4, *p* < 0.001; condition × age interaction for GluR2, *F*_4,45_ = 5.002, *p* = 0.002; main effect of age for PSD-95, *F*_2,45_ = 36.418, *p* < 0.001; Fig. 2B,D). In the IL, neither aging nor fear conditioning affected the expression of PKC isoforms, GluR1, GluR2, or PSD-95 (all *p* > 0.05; Fig. 2C,E). These results indicate that the impairment in LTM retention in aged rats was associated with a decrease in PKMζ expression in the PrL.

**Figure 3:**
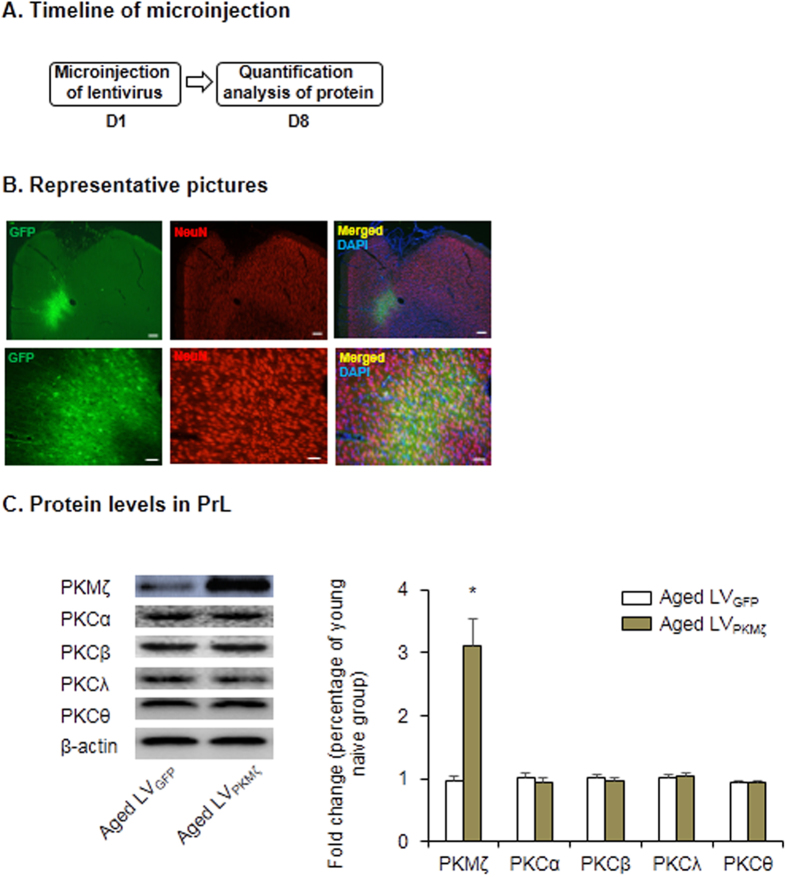
PKMζ was overexpressed in the PrL. (**A**) Experimental timeline for PKMζ detection. (**B**) Representative pictures of a coronal section of double-labeling for GFP (green) and NeuN (red), counterstained with DAPI (blue) 7 days after microinjection. Scale bar: the top row, 200 μm; the bottom row, 50 μm. (**C**) PKMζ expression in the PrL of rats microinfused with lentiviral vector expressing PKMζ was quantified using Western Blot. *p < 0.05, compared with LV_GFP_ group; n = 9 per experimental condition.

**Figure 4:**
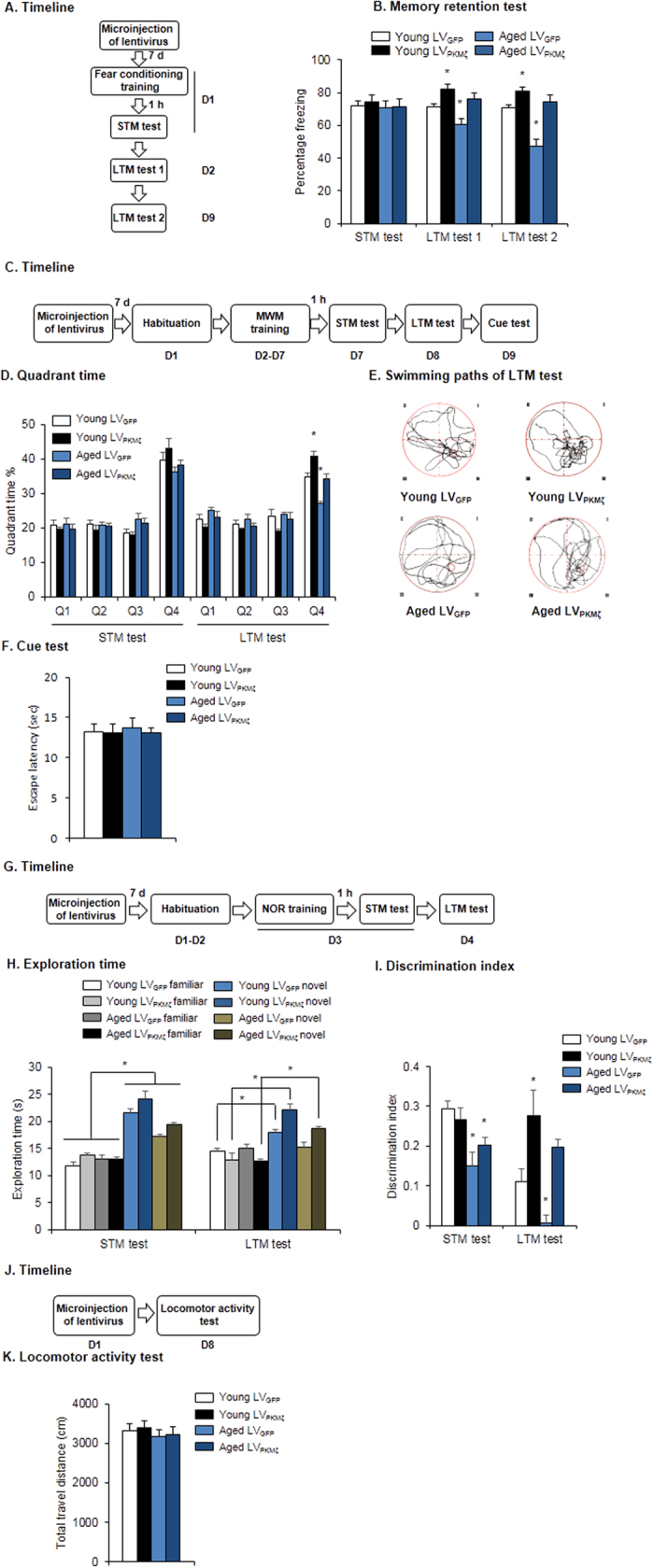
Overexpression of PKMζ in the PrL improved memory retention in aged rats. (**A,B**) PKMζ overexpression in the PrL improved memory retention of fear conditioning in aged rats. (**A**) Experimental timeline for lentivirus microinjection and fear conditioning. (**B**) Freezing scores during STM test, LTM test 1 and memory persistence test (LTM test 2). *p < 0.05, compared with young LV_GFP_ group, n = 8–10 per experimental condition. **(C-F)** PKMζ overexpression in the PrL improved memory retention in aged rats in the MWM task. (**C**) Experimental timeline for lentivirus microinjection and the MWM task. (**D**) Quadrant time during STM and LTM probe tests. *p < 0.05, compared with young LV_GFP_ group. *n* = 9–10 per experimental condition. (**E**) Swimming paths during the LTM probe test. (**F**) Cue test. (**G–I**) PKMζ overexpression improved memory retention in aged rats in the NOR task. (**G**) Experimental timeline for lentivirus microinjection and the NOR task. **(H)** Exploration time during the STM and LTM tests. *p < 0.05, compared with age-matched familiar group. *n* = 8–9 per experimental condition. (**I**) Discrimination index. *p < 0.05, compared with young LV_GFP_ group. *n* = 8–9 per experimental condition. (**J–K**) Locomotor activity was unaffected by PKMζ overexpression. (**J**) Experimental timeline for lentivirus microinjection and locomotor activity test. (**K**) Total travel distance during the locomotor activity test. *n* = 8-10 per experimental condition.

We then assessed whether PKMζ overexpression in the PrL could restore LTM in aged rats. Lentiviral vectors that contained *PKMζ* (LV_PKMζ_) were constructed and microinfused into the PrL in aged rats to selectively overexpress PKMζ. First, we confirmed the efficiency of virus transfection in the PrL in aged rats. One week after infusion, rats were euthanized for immunofluorescence and Western blot assays (Fig. 3A). The expression of plasmid GFP was verified, and we double-labeled PrL tissue sections with the neuronal marker NeuN. The results revealed strong colocalization of plasmid GFP and NeuN signals in the PrL (Fig. 3B). Furthermore, PKMζ expression in the PrL was significantly increased in aged LV_PKMζ_ group (one-way ANOVA, *F*_1, 16_ = 15.061, p = 0.001, Fig. 3C), but no variation was found in the expression of other PKC isoforms. Aged and young rats that received LV_PKMζ_ or LV_GFP_ infusion were subjected to contextual fear conditioning and underwent tests 1 h, 1 day, and 8 days later (Fig. 4A). After the intra-PrL LV_PKMζ_ infusion, we found that LTM in aged rats was enhanced to the level of young rats that were injected with LV_GFP_. Young rats that were injected with LV_PKMζ_ exhibited a stronger fear response compared with young rats that were injected with LV_GFP_ (two-way ANOVA, main effect of group, *F*_1,30_ = 4.289, *p* = 0.047; main effect of vector, *F*_1,30_ = 11.258, *p* = 0.002; Fig. 4B, middle column). This fear-enhancing effect lasted for at least 8 days in both the aged LV_PKMζ_ group and young LV_PKMζ_ group (two-way ANOVA, main effect of group, *F*_1,30_ = 10.845, *p* = 0.003; main effect of vector, *F*_1,30_ = 16.414, *p* < 0.001; Fig. 4B, right column). Intra-PrL LV_PKMζ_ infusion had no effect on STM (*p* > 0.05; Fig. 4B, left column).

In the MWM and NOR test, we also found that LTM in aged rats was restored after PKMζ overexpression (Fig. 4C–I). In the MWM (Fig. 4C), all four groups presented similar performance in the STM test. In the LTM test, the time spent in the target quadrant increased in aged rats after intra-PrL LV_PKMζ_ infusion to similar levels as in young LV_GFP_ rats. An enhancing effect of intra-PrL LV_PKMζ_ infusion on the time spent in the target quadrant was also observed in young rats (multi-way ANOVA, main effect of quadrant, *F*_3,140_ = 52.548, *p* < 0.001; age × quadrant interaction, *F*_3,140_ = 7.326, *p* < 0.001; vector × quadrant interaction, *F*_3,140_ = 6.883, *p* < 0.001; Fig. 4D). The swimming paths also showed that both young LV_GFP_ rats and aged LV_PKMζ_ rats exhibited higher preference for the target quadrant compared with aged LV_GFP_ rats, and young LV_PKMζ_ rats exhibited the highest preference (Fig. 4E). All of the rats spent similar time reaching the visible platform during the cue test (*p* > 0.05; Fig. 4F). In the NOR test (Fig. 4G), all of the groups presented preference for the novel object during the STM test, whereas young LV_GFP_ rats, young LV_PKMζ_ rats, and aged LV_PKMζ_ rats but not aged LV_GFP_ rats exhibited preference for the novel object during the LTM test (multi-way ANOVA, main effect of object, *F*_1,62_ = 41.962, *p* < 0.001; age × object interaction, *F*_1,62_ = 4.799, *p* = 0.032; vector × object interaction, *F*_1,62_ = 15.371, *p* < 0.001; Fig. 4H). The discrimination index was significantly lower in the aged LV_GFP_ group and aged LV_PKMζ_ group in the STM test (two-way ANOVA, main effect of group, *F*_1,31_ = 9.306, *p* = 0.005; Fig. 4I). The young LV_PKMζ_ group had a higher discrimination index than the young LV_GFP_ group during the LTM test, and the aged LV_GFP_ group had a lower discrimination index (two-way ANOVA, main effect of group, *F*_1,31_ = 4.226, *p* = 0.036; main effect of vector, *F*_1,31_ = 11.992, *p* = 0.002; Fig. 4I). Locomotor activity was unaffected (*p* > 0.05; Fig. 4J,K). Altogether, these results indicate that the decrease in prelimbic PKMζ is responsible for the aging-related impairment in LTM.

### Aged rats lacked learning-related changes in unmethylated *PKMζ* DNA

**Figure 5:**
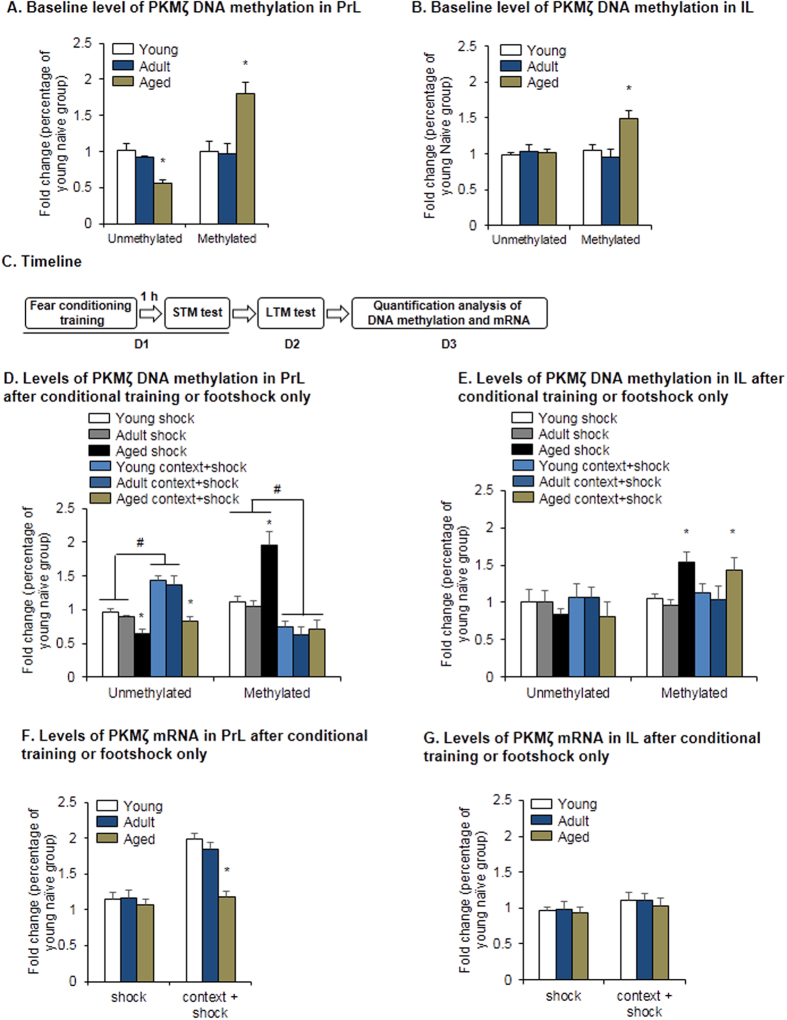
Aged rats lacked learning-related changes in unmethylated *PKMζ* DNA. (**A**) Baseline level of *PKMζ* DNA methylation in the PrL. *p < 0.05, compared with young group. *n* = 5 per experimental group. (**B**) Baseline level of *PKMζ* DNA methylation in the IL. *p < 0.05, compared with young group. *n* = 5 per experimental group. (**C**) Experimental timeline for detection of *PKMζ* DNA methylation and mRNA. (**D**) Levels of methylated and unmethylated *PKMζ* DNA in the PrL after conditioning training or footshock only. *p < 0.05, compared with manipulation-matched young group; ^#^*p* < 0.05, compared with age-matched shock group. *n* = 6–7 per experimental condition. (**E**) Levels of methylated and unmethylated *PKMζ* DNA in the IL after conditioning training or footshock only. *p < 0.05, compared with manipulation-matched young group. *n* = 6–7 per experimental condition. (**F**) Level of *PKMζ* mRNA in the PrL after conditioning training or footshock only. *p < 0.05, compared with manipulation-matched young group. *n* = 6–7 per experimental condition. **(G)** Level of *PKMζ* mRNA in the IL after conditioning training or footshock only.

We next explored why the expression of PKMζ protein was decreased in aged rats. DNA methylation is critical for the regulation of gene expression^13^,^14^, and abnormal DNA methylation affects cognitive abilities in aged animals^15^,^17^. Thus, we assessed whether the aberrant methylation of CpG island sites in the promoter regions of the *PKMζ* gene in the PrL contributed to decreases in PKMζ expression in aged rats. We first assessed baseline levels of methylated and unmethylated *PKMζ* DNA in the PrL and IL in rats at different ages. We found that the level of unmethylated *PKMζ* DNA in the PrL decreased in aged rats compared with young and adult rats (one-way ANOVA, *F*_2,12_ = 9.10, *p* = 0.004), whereas the level of methylation of *PKMζ* DNA increased (one-way ANOVA, *F*_2,12_ = 6.97, *p* = 0.01; Fig. 5A). In the IL, only the methylation level increased in aged rats (one-way ANOVA, *F*_2,12_ = 5.53, *p* = 0.02; Fig. 5B). DNA methylation has been found to be critical for the formation and long-lasting maintenance of memory^13^,^14^. Thus, we assessed alterations in the DNA methylation of *PKMζ* after fear conditioning. Rats at different ages (3, 9, and 24 months) underwent contextual fear conditioning or electric shock only. One day after the LTM test, we detected the levels of methylated PKMζ DNA and PKMζ mRNA in PrL and IL (Fig. 5C). In the PrL, we found that fear conditioning increased the level of unmethylated *PKMζ* DNA and decreased the level of methylated *PKMζ* DNA in young and adult rats. Aged rats exhibited a decrease in the level of methylated DNA but no change in the level of unmethylated DNA (two-way ANOVA; significant effect of conditioning, *F*_1,34_ = 19.287, *p* < 0.001, for unmethylated DNA; *F*_1,34_ = 11.70, *p* = 0.002, for methylated DNA; significant effect of age, *F*_2,34_ = 11.23, *p* < 0.001, for unmethylated DNA, *F*_2,34_ = 4.48, *p* = 0.019, for methylated DNA; Fig. 5D). In the IL, fear conditioning did not affect the level of methylated and unmethylated *PKMζ* DNA (*p* > 0.05; Fig. 5E). *PKMζ* mRNA levels increased in the PrL after fear conditioning in young and adult rats but not in aged rats (two-way ANOVA; main effect of age, *F*_2,32_ = 3.82, *p* < 0.001; main effect of conditioning, *F*_1,32_ = 31.30, *p* < 0.001; Fig. 5F). In the IL, no significant difference was found in the level of *PKMζ* mRNA among rats at different ages (*p* > 0.05; Fig. 5G). Overall, these results indicate that the decrease in methylated DNA and increase in unmethylated DNA at promoter CpG islands during LTM retention were correlated with increases in the transcription and translation of *PKMζ* in young and adult rats but not aged rats. The lack of learning-related changes in unmethylated *PKMζ* DNA might mediate impaired memory retention in aged rats.

### Environmental enrichment improved memory retention in aged rats and down-regulated methylated *PKMζ* DNA in the PrL

**Figure 6:**
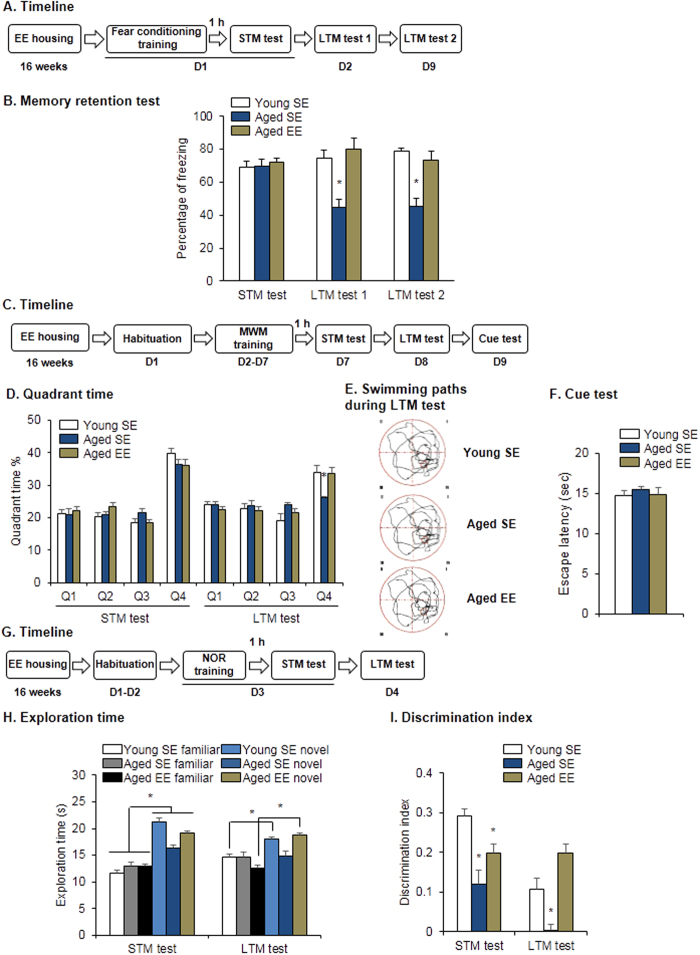
Environmentally enriched housing improved memory retention in aged rats. (**A,B**) Environmentally enriched housing rescued the impairment in memory retention of fear conditioning in aged rats. (**A**) Experimental timeline for environmental enrichment and fear conditioning. (**B**) Freezing scores during the STM test, LTM test 1, and remote memory test (LTM test 2). *p < 0.05, compared with young SE group. *n* = 8–10 per experimental condition. (**C–F**) Environmentally enriched housing improved memory retention in aged rats in the MWM task. (**C**) Experimental timeline for environmental enrichment and the MWM task. (**D**) Quadrant time during the STM and LTM probe tests. *p < 0.05, compared with young SE group. *n* = 8–10 per experimental condition. (**E**) Swimming paths during LTM probe test. (**F**) Cue test. (**G–I**) Environmentally enriched housing improved memory retention in aged rats in the NOR task. (**G**) Experimental timeline for environmental enrichment and the NOR task. (**H**) Exploration time during the STM and LTM tests. *p < 0.05, compared with age-matched familiar group. *n* = 8–10 per experimental condition. (**I**) Discrimination index. *p < 0.05, compared with young SE group. *n* = 8–10 per experimental condition. EE, environmentally enriched housing; SE, standard-environment housing.

Environmentally enriched housing improves memory retention in aged rats, which involves different molecular pathways and brain areas^27^,^28^. We investigated whether long-term environmental enrichment improved memory retention in aged rats by altering the regulation of *PKMζ* transcription in the mPFC. We first assessed whether environmentally enriched housing was sufficient to improve LTM in aged rats. Aged rats with 4 months of environmentally enriched housing or standard-environment housing were subjected to fear conditioning. STM, LTM, and the long-term persistence of memory were tested 1 h, 24 h, and 8 days later (Fig. 6A). LTM (one-way ANOVA, *F*_2,25_ = 4.70, *p* = 0.02) and memory persistence (one-way ANOVA, *F*_2,25_ = 7.96, *p* = 0.002; Fig. 6B) significantly increased in aged rats after environmentally enriched housing to the levels of young rats after standard-environment housing.

In the MWM and NOR test, environmental enrichment also improved LTM in aged rats (Fig. 6C–I). In the MWM (Fig. 6C), all three groups presented similar performance in the STM test. In the LTM test, the time spent in the target quadrant (two-way ANOVA, main effect of quadrant, *F*_3,100_ = 19.025, *p* < 0.001; Fig. 6D) increased in aged rats after environmentally enriched housing to similar levels as in young rats. This increase in the time spent in the target quadrant was not observed in aged rats after standard-environment housing. The swimming paths also showed that aged rats housed in enriched environment and young rats had higher preference for the target quadrant compared with aged rats housed in standard environment (Fig. 6E). All of the rats spent similar time reaching the visible platform during the cue test (*p* > 0.05; Fig. 6F). In NOR test (Fig. 6G), all groups had higher preference for the novel object during the STM test. During the LTM test, like young rats, aged rats exhibited significant preference for the novel object after environmentally enriched housing (two-way ANOVA, main effect of object, *F*_1,50_ = 23.027, *p* < 0.001; interaction effect of group × object, *F*_2,50_ = 6.617, *p* = 0.003; Fig. 6H). Discrimination index was significantly lower in aged rats housed in standard environment in both STM test (one-way ANOVA, *F*_2,25_ = 6.199, *p* = 0.007) and LTM test (one-way ANOVA, *F*_2,25_ = 9.591, *p* = 0.001; Fig. 6I). These results indicate that environmentally enriched housing restored cognitive performance in aged rats.

**Figure 7:**
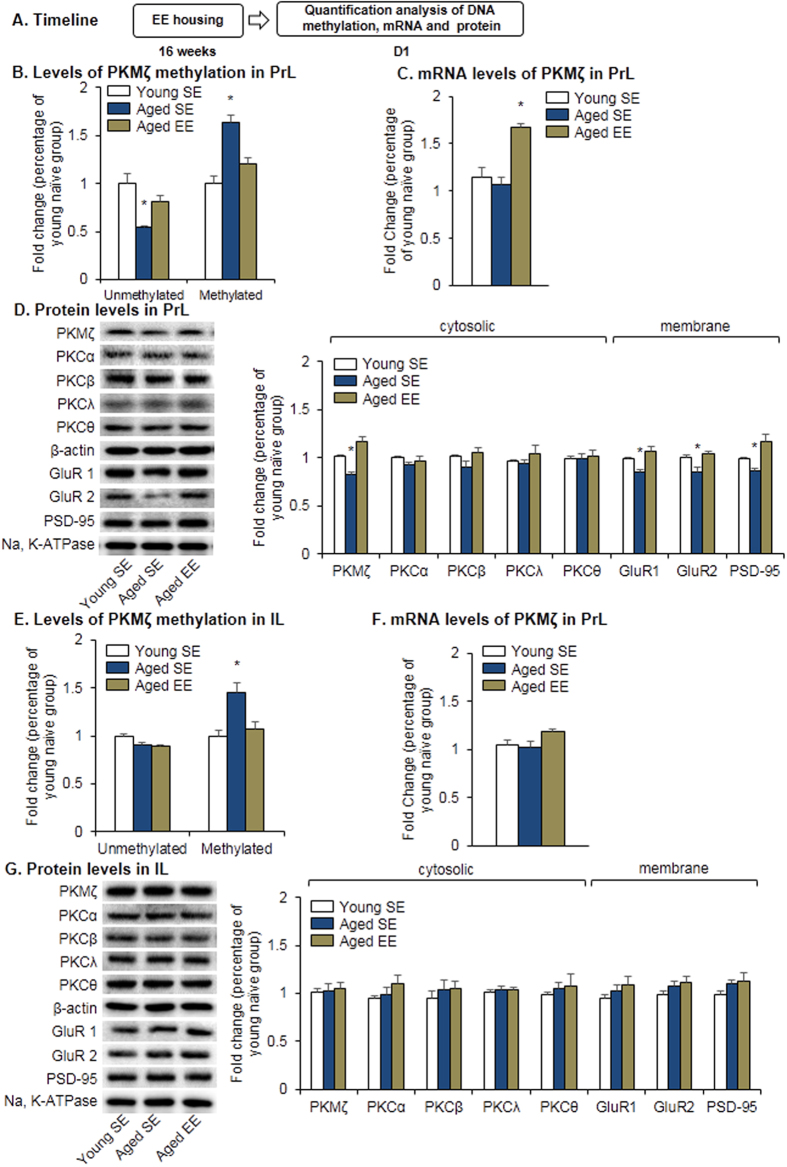
Environmental enrichment down-regulated methylated *PKMζ* DNA in the PrL in aged rats. (**A**) Experimental timeline for the detection of *PKMζ* DNA methylation, mRNA, and protein levels. (**B,E**) Levels of methylated and unmethylated *PKMζ* DNA in the PrL (**B**) and IL (**E**) after environmental enrichment and fear conditioning. *p < 0.05, compared with young SE group. (**C,F**) Level of *PKMζ* mRNA in the PrL (**C**) and IL (**F**) in different age groups after environmentally enriched housing or standard-environment housing. (**D,G**) Cytosolic and membrane protein levels and representative Western blots in the PrL (**D**) and IL (**G**) after environmentally enriched housing. Levels of membrane proteins including GluR1, GluR2 and PSD-95 were normalized to Na, K-ATPase and levels of cytosolic proteins like PKMζ, PKCα, PKCβ, PKCλ, and PKCθ were normalized to β-actin. *p < 0.05, compared with young SE group. *n* = 8–10 per experimental condition. EE, environmentally enriched housing; SE, standard-environment housing.

We next assessed whether environmentally enriched housing rescues aberrant hypermethylation in promoter regions of the *PKMζ* gene in the PrL in aged rats. After 4 months of environmentally enriched or standard-environment housing, brains were harvested from aged and young rats for subsequent determination of the levels of *PKMζ* DNA methylation and mRNA (Fig. 7A). We found that environmental enrichment decreased the level of methylated DNA (one-way ANOVA, *F*_2,23_ = 11.98, *p* < 0.001) and increased the level of unmethylated DNA (one-way ANOVA, *F*_2,23_ = 6.42, *p* = 0.006; Fig. 7B) in the PrL in aged rats. The mRNA level of *PKMζ* was also increased in aged rats that received environmental enrichment (one-way ANOVA, *F*_2,17_ = 9.39, *p* = 0.002; Fig. 7C). We also measured the expression of different isoforms of PKC (i.e., PKMζ, PKCα, PKCβ, PKCλ, and PKCθ), GluR1, GluR2, and PSD-95 in the PrL and IL after environmentally enriched or standard-environment housing. Environmental enrichment significantly increased the cytosolic expression of PKMζ (one-way ANOVA, *F*_2,24_ = 5.27, *p* = 0.013) and the membrane expression of GluR1 (one-way ANOVA, *F*_2,24_ = 6.20, *p* = 0.007), GluR2 (one-way ANOVA, *F*_2,24_ = 5.94, *p* = 0.008), and PSD-95 (one-way ANOVA, *F*_2,24_ = 5.57, *p* = 0.01) in aged rats (Fig. 7D). Environmental enrichment had no significant effect on the cytosolic expression of the other isoforms of PKC, including PKCα, PKCβ, PKCλ, and PKCθ (all *p* > 0.05; Fig. 7D). In the IL, environmental enrichment had no effect on the levels of unmethylated *PKMζ* DNA, *PKMζ* mRNA, or protein expression of GluR1, GluR2, or PSD-95 (*p* > 0.05; Fig. 7E–G). Overall, these results indicate that environmental enrichment induced a transcriptional program that led to the activation of plasticity-associated genes in the PrL in aged rats. Environmental enrichment down-regulated methylated DNA of PKMζ in the PrL and restored memory retention in aged rats.

### Dominant-negative mutant of PKMζ reversed the effect of environmentally enriched housing on memory retention in aged rats

**Figure 8:**
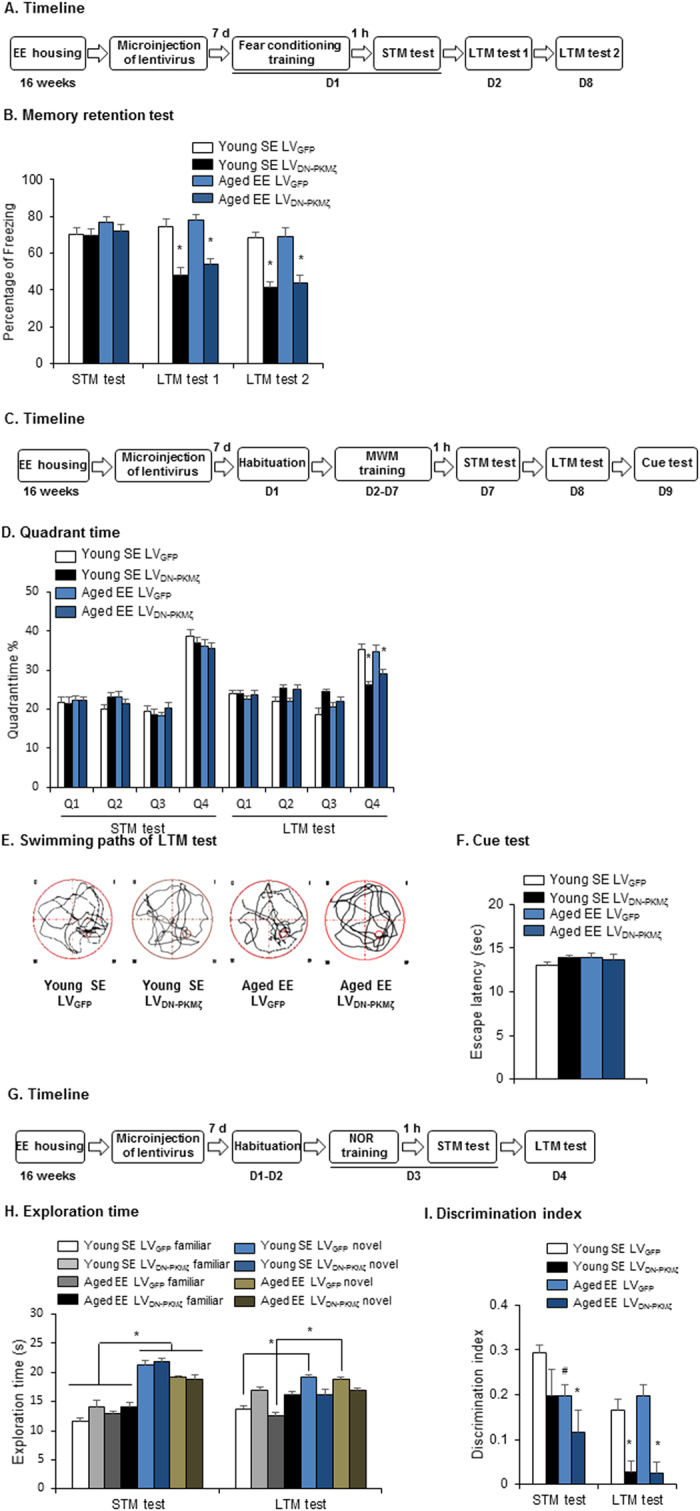
Inactivation of PKMζ reversed the potentiating effect of environmentally enriched housing on memory retention in aged rats. (**A,B**) Microinjection of LV_DN-PKMζ_ in the PrL reversed the effect of environmentally enriched housing on memory retention of fear conditioning in aged rats. (**A**) Experimental timeline for lentivirus microinjection and fear conditioning. (**B**) Freezing scores during the STM test, LTM test 1, and remote memory test (LTM test 2) after lentivirus microinfusion. *p < 0.05, compared with age-matched group. *n* = 8–10 per experimental condition. (**C–F**) Microinjection of LV_DN-PKMζ_ in the PrL reversed the potentiating effect of environmentally enriched housing on memory retention in aged rats in the MWM task. (**C**) Experimental timeline for lentivirus microinjection and the MWM task. (**D**) Quadrant time during the STM and LTM probe tests. *p < 0.05, compared with age-matched LV_GFP_ group. *n* = 8–11 per experimental condition. (**E**) Swimming paths during the LTM probe test. (**F**) Cue test. (**G–I**) Microinjection of LV_DN-PKMζ_ in the PrL reversed the potentiating effect of environmentally enriched housing on memory retention in aged rats in the NOR task. (**G**) Experimental timeline for lentivirus microinjection and the NOR task. (**H**) Exploration time during the STM and LTM tests. *p < 0.05, compared with manipulation-matched familiar group. *n* = 8–10 per experimental condition. (**I**) Discrimination index. *p < 0.05, compared with age-matched LV_GFP_ group ^#^*p* < 0.05, compared with young SE LVGFP group. *n* = 8–10 per experimental condition.

Lastly, we investigated the specific role of PKMζ in the effect of environmentally enriched housing on cognition. We microinfused a lentiviral vector that expressed mutant PKMζ (LV_DN-PKMζ_) in the PrL to suppress the activity of prelimbic PKMζ^23^. Aged rats received 4 months of environmentally enriched housing and then received infusions of LV_DN-PKMζ_ or LV_GFP_ in the PrL. All of the rats were subjected to fear conditioning, STM, LTM, and memory persistence tests (Fig. 8A). The potentiating effects of environmental enrichment on LTM (two-way ANOVA, *F*_1,31_ = 13.819, *p* = 0.001; Fig. 8B) and memory persistence (two-way ANOVA, *F*_1,31_ = 18.441, *p* < 0.001; Fig. 8B) decreased in both young rats and aged rats that received intra-PrL LV_DN-PKMζ_ infusions. We obtained similar results in the MWM and NOR test. Suppression of the activity of PKMζ disrupted the potentiating effect of environmental enrichment on LTM (Fig. 8C–I). In the MWM (Fig. 8C), all four groups presented similar performance in the STM test. In the LTM test, the time spent in the target quadrant (multi-way ANOVA, main effect of quadrant, *F*_3,132_ = 35.363, *p* < 0.001; vector × quadrant interaction, *F*_3,132_ = 12.4595, *p* < 0.001; Fig. 8D) decreased in both young rats and aged rats that received intra-PrL LV_DN-PKMζ_ infusions. The swimming paths also showed that both young rats that were housed in a standard environment and aged rats that were housed in an enriched environment presented lower preference for the target quadrant after intra-PrL LV_DN-PKMζ_ infusions (Fig. 8E). All of the rats spent similar time reaching the visible platform during the cue test (*p* > 0.05; Fig. 8F). In the NOR test (Fig. 8G), all of the groups presented preference for the novel object in the STM test. In the LTM test, young rats that were housed in a standard environment and aged rats that were housed in an enriched environment exhibited significantly lower preference for the novel object when they received intra-PrL LV_DN-PKMζ_ infusions (multi-way ANOVA, main effect of object, *F*_1,58_ = 33.928, *p* < 0.001; vector × object interaction, *F*_1,58_ = 33.510, *p* < 0.001; Fig. 8H). Additionally, the effects of environmental enrichment on the discrimination index decreased in aged rats that received intra-PrL LV_DN-PKMζ_ infusions in the STM test (two-way ANOVA, main effect of vector, *F*_1,29_ = 4.977, *p* = 0.034) and LTM test (two-way ANOVA, main effect of vector, *F*_1,29_ = 38.671, *p* < 0.001; Fig. 8I). Young rats that were housed in a standard environment also had a lower discrimination index in both the STM and LTM tests after LV_DN-PKMζ_ infusions. Overall, inactivating PKMζ in the PrL reversed the beneficial effect of environmental enrichment on cognitive ability in aged rats.

## Discussion

Altogether, the aberrant hypermethylation of *PKMζ* DNA in the PrL contributed to aging-related impairment in memory retention. Associative learning decreased the level of methylated *PKMζ* DNA in all age groups during LTM retention, and increased the level of unmethylated *PKMζ* DNA only in young and adult rats but not in aged rats. Environmentally enriched housing upregulated PKMζ protein and unmethylated *PKMζ* DNA, downregulated methylated *PKMζ* DNA expression, and consequently improved LTM retention in multiple behavior models, and these effects were reversed by microinfusion of DN mutant of PKMζ in the PrL.

Previous research showed that PKMζ regulates memory retention in multiple learning tasks, including MWM, T-maze, contextual and auditory fear conditioning, recognition memory, conditioned inhibitory avoidance, drug reward, and aversive memory, which involve the hippocampus, basolateral amygdala, and mPFC^20^,^21^,^23^,^25^,^29^. Several studies argued that PKMζ might not be necessary for memory maintenance. Lee *et al.*^30^ used knockout mice with targeted deletion of exon 9 of the catalytic domain of PKMζ and found normal learning and memory in a cued fear conditioning paradigm, the NOR test, and a cocaine-induced conditioned place preference test. Volk *et al.*^31^ used knockout mice with targeted deletion of exon 11 of the catalytic domain of PKMζ and observed normal hippocampal synaptic plasticity, normal acquisition and retention of fear conditioning, and normal MWM performance. Zeta inhibitory peptide (ZIP) has been used in many studies to inhibit the activity of PKMζ, but such inhibition is not specific to PKMζ^21^,^32^,^33^. Some studies independent of ZIP have also supported a role for PKMζ in memory and synaptic plasticity^23^,^34^. For example, overexpression of PKMζ in the neocortex enhances long-term memory of conditioned taste aversion, while DN mutant of PKMζ impairs memory^23^. On the other hand, PKMζ knockout may disrupt the balance of PKMζ expression in the brain, leading to developmental changes in the functions of other genes^35^,^36^. In the present study, PKCι/λ did not increase after the LTM test or environmental enrichment in aged rats. Therefore, we speculate that PKCι/λ may compensate for the loss of function of PKMζ in PKC/PKMζ knockout mice but not in normal animals, including aged rats, in which PKMζ is physiologically downregulated with advancing age. Indeed, compared with aged rhesus monkeys, young monkeys that displayed faster acquisition of the delayed nonmatching-to-sample (DNMS) task and more accurate recognition memory exhibited a higher proportion of dendritic spines that co-express GluA2 and PKMζ^37^. Overall, our data suggest a causal relationship between aging-induced cognitive impairment and the transcriptional regulation of PKMζ expression.

DNA methylation is one of the most prominent covalent DNA modifications that involve the conversion of cytosines at CpG dinucleotides to 5-methylcytosine. The increase in the level of DNA methylation by DNA methyltransferases results in transcriptional silencing and the loss of gene function, either because of the inhibition of binding of transcription factors or through the recruitment of proteins that contain methyl-binding domains^38^,^39^. The degree of DNA methylation in the brain increases during aging^40^,^41^. We found that aged rats exhibited increases in the baseline level of methylated *PKMζ* DNA and decreases in unmethylated *PKMζ* DNA compared with young and adult rats. These age-related changes might result in deficits in learning-related transcriptional activation, leading to memory impairments. After fear conditioning, the levels of methylated *PKMζ* DNA in PrL decreased in all age groups, whereas the levels of unmethylated *PKMζ* DNA increased in young and adult rats, but not in aged rats. We speculate that aged rats might exhibit impairment in experience-dependent DNA demethylation, in which 5-methylcytosine might not be able to be fully converted to cytosines and some cytosine derivatives might be generated, including 5-hydroxymethylcytosines (5hmC)^42^, 5-formylcytosine and 5-carboxylcytosine^43^, via passive and/or active mechanisms of DNA demethylation. Further research is needed to determine the role of DNA hydroxylation and decarboxylation in learning and memory.

The environmental enrichment procedure is considered a noninvasive intervention for aging-related memory impairment^27^,^28^,^44^,^45^,^46^. Environmental enrichment restores learning ability by elevating the acetylation of histones H3 and H4^47^. In the present study, we found that an increase in endogenic PKMζ expression in the PrL is required for the potentiating effect of environmental enrichment on cognitive function in aged rats, and environmental enrichment increased the level of unmethylated *PKMζ* DNA and decreased the level of methylated *PKMζ* DNA in the PrL in aged rats. However, the mechanisms that underlie the environmental enrichment-induced increase in the level of unmethylated *PKMζ* DNA are unknown, and both passive and active mechanisms of DNA demethylation may be involved. Recent research has shown that environmental enrichment improved learning and memory in aged mice by modulating the dynamics of 5hmC in the hippocampus^48^. Thus, evidence indicates that the absence or reduction of DNA methyltransferases leads to passive DNA demethylation^49^,^50^. Other transcriptional regulators that are involved in the regulation of synaptic plasticity, such as methyl-CpG-binding protein 2, may also be recruited^39^,^51^.

In conclusion, we found that the hypermethylation of *PKMζ* might impair the retention of LTM in aged rats, and environmental enrichment reversed cognitive impairment in aged rats probably by facilitating *PKMζ* DNA demethylation in the PrL. These findings extend the knowledge of PKMζ signaling in memory retention in aged animals and provide insights into the epigenetic mechanisms that underlie the potential therapeutic effect of environmental enrichment on memory decline.

## Materials and Methods

### Subjects

Male Sprague-Dawley rats [young rats (young): 3 months old upon arrival, weighing 300–320 g; adult rats (adult): 9 months old upon arrival, weighting 480–500 g; aged rats (aged): 20 months old upon arrival, weighting 600–620 g, housed under environmentally enriched or standard-environment housing conditions for an additional 4 months after arrival in the laboratory] were obtained from the Laboratory Animal Center, Peking University Health Science Center. The animals were housed under conditions of a 12 h/12 h light/dark cycle (lights on at 8:00 AM) at 23 ± 2 °C and 50% ± 5% humidity. Food and water were provided *ad libitum*. All of the experimental procedures were performed with approval from the Biomedical Ethics Committee for Animal Use and Protection of Peking University and in accordance to the National Institutes of Health Guide for the Care and Use of Laboratory Animals.

### Surgery

The rats were anesthetized with sodium pentobarbital (50 mg/kg, i.p.). Guide cannulae (23 gauge; Plastics One, Roanoke, VA, USA) were bilaterally implanted 1 mm above the PrL. The coordinates were modified from previous studies^20^ and were the following: anterior/posterior, +2.9 mm; medial/lateral, ±2.3 mm; dorsal/ventral, −3.0 mm. The cannulae were placed at a 16° angle toward the midline to avoid penetration of the lateral ventricle. The rats were allowed to recover for 7 days after surgery.

### Lentiviral vector construction and injection

Construction and injection of the lentiviral vectors were based on our previous studies^52^,^53^. Two vector plasmids, pSinRep5-pPKMζ-Ires-ZsGreen and pSinRep5-pPKMζ-K281W-Ires-ZsGreen, were provided by Prof. Todd Sacktor (SUNY Downstate Medical Center, Brooklyn, NY, USA). Vector plasmids were constructed for the production of lentiviruses that express PKMζ or a dominant-negative (DN) mutant of PKMζ (PKMζ-K281W, amino acid sequence number as in PKCζ). An IRES structure was used to separately overexpress GFP and PKMζ or DN mutant of PKMζ. *PKMζ* was amplified by polymerase chain reaction (PCR) from the vectors and subcloned into the GV208 vector using *BamH*I and *Age*I restriction sites. All of the vectors contained the green fluorescence protein (GFP) coding sequence. All of the vectors were then transfected into human embryonic kidney 293 cells. Approximately 48 h post-transfection, cells were harvested, purified by centrifugation, and stored at −80 °C. The virus injection protocols were based on previous studies^23^,^52^. The rats were anesthetized with sodium pentobarbital (60 mg/kg, i.p.). Hamilton syringes were connected to 30-gauge injectors (Plastics One, Roanoke, VA, USA) that reached 1 mm below the guide cannula. The LV_PKMζ_, LV_DN-PKMζ_, and LV_GFP_ (1 × 10^9^ viral genomes, dissolved in phosphate-buffered saline [PBS]) were injected into the PrL with 10 μl Hamilton syringes that were connected via polyethylene-50 tubing to 30-gauge injectors. The lentiviral vectors (1 μl to each side) were infused bilaterally over 10 min, and the injection needle was kept in place for additional 5 min to allow for diffusion.

### Contextual fear conditioning

The procedure for contextual fear conditioning was modified from previous studies^54^. On the first day, the conditioning group were placed in the training chamber and allowed to explore the chamber for 2 min, after which they received three electric footshocks (0.85 mA, 1 s) with 2-min intervals. The rats were then allowed to explore the conditioning chamber for an additional 1 min. The shock group only received three consecutive 1-s footshocks (0.85 mA), and were returned immediately to their homecage before they were able to form an association with the context. Short-term memory (STM) and long-term memory (LTM) were tested 1 and 24 h after conditioning, respectively. Remote memory was tested 8 days after conditioning in the rats that received lentivirus microinfusions, oligonucleotide microinfusions, and environmental enrichment. All of the memory tests were conducted by exposing the rats to the conditioning chamber for 5 min without footshock.

### Morris water maze

The MWM procedure was based on a previous study^55^. A circular black-painted swimming pool (diameter: 1.5 m) was divided into four equal quadrants (Q1, Q2, Q3, and Q4) and a black escape platform was placed 2 cm below the water surface in the center of Q4. On day 1, the rats were habituated to the swimming pool. During training, the rats underwent three trials per day at 5-min intertrial intervals to find the hidden platform for 6 consecutive days. The STM and LTM tests were conducted 1 and 24 h after the last training day, during which the platform was removed. The average of three trials was taken.

Sensorimotor ability was assessed by a cue test 24 h after the LTM test, during which the time spent reaching a visible black platform extending 2 cm above the water surface was recorded. The cue test consisted of four trials at 60-s intertrial intervals and the average of the four trials was taken.

### Novel object recognition

The NOR procedure was modified from a previous study^7^. The test apparatus was two identical isolated black open field boxes (85 cm length × 85 cm width × 55 cm height). The discriminated objects were made of glass or plastic. Each object was available in four identical copies, and the weights were such that a rat could not move the objects. The objects varied in height from 10 to 15 cm and width from 8 to 10 cm. Habituation to the test apparatus consisted of two daily 10-min sessions, in which the animals were allowed to freely explore the open field box. The NOR test was conducted on the third and fourth days. Each test day began with a 3-min training session (i.e., A/A session with identical objects), followed 1 and 24 h later by a 3-min test session (i.e., A/B session with dissimilar objects). The time that a rat spent exploring each object was recorded. A discrimination index was calculated in each A/B trial and defined as the difference in time spent exploring the novel and familiar objects divided by the total exploration time for both objects.

### Environmental enrichment

Aged rats were randomly assigned to and housed in enriched or standard conditions for 16 weeks. Young rats were housed in standard conditions as a control. Environmentally enriched rats were housed in groups of 12 in large cages (100 cm length × 100 cm width × 90 cm height) with a plastic floor and wire mesh walls. Inside the cage were two running wheels, two rodent dwellings, a plastic tube, two large plastic ramps that connected the floor, and two platforms approximately 35 cm and 70 cm above the floor. Additional objects (e.g., wooden and metallic objects, small toys and balls, etc.) were placed in the cages^56^. The objects in this cage were moved or changed every week. The rats that were housed in a standard environment were reared in groups of three to five in plastic cages (45 cm length × 35 cm width × 25 cm height) and had no exposure to toys or running wheels.

### Locomotor activity test

Locomotor activity was measured with an automated video tracking system as described in our previous study^57^. Eight identical black Plexiglas chambers (40 cm × 40 cm × 65 cm) were connected to a computer that recorded movement trails. Locomotor activity was analyzed using DigBehv analysis software and is expressed as the total distance traveled during the 30-min test.

### Tissue sample preparation

The procedure was based on our previous studies^54^,^58^. All of the rats were decapitated 1 day after the LTM test of fear conditioning or 1 day after environmentally enriched housing. The brains were rapidly extracted, frozen in −60 °C N-hexane, and transferred to a −80 °C freezer. Bilateral tissue punches (16 gauge) of the PrL and IL were taken from 1-mm-thick coronal sections in a freezing cryostat (−20 °C) for subsequent analysis.

### Western blot assays

The Western blot assays were based on our previous studies^54^,^58^,^59^. The tissue punches were placed in ice-cold homogenization buffer (0.32 M sucrose, 4 mM HEPES, 1 mM EDTA, 1 mM EGTA, and protease/phosphatase inhibitor cocktail, pH 7.4). After being homogenized, the homogenate was centrifuged at 1000 × *g* for 10 min at 4 °C to obtain the pellet (P1) that contained nuclei and large debris. The supernatant (S1) was again centrifuged at 10,000 × *g* for 30 min at 4 °C to generate a crude synaptosomal fraction (P2) and supernatant (S2). The crude synaptosomal membrane pellet (P2) was hypoosmotically lysed and centrifuged at 25,000 × *g* for 30 min at 4 °C to generate the synaptosomal membrane fraction (LP1). LP1 was resuspended in HEPES-lysis buffer (50 mM HEPES, 1 mM EDTA, 1 mM EGTA, and protease/phosphatase inhibitor cocktail, pH 7.4). The protein concentrations of all of the samples (S2 and LP1) were determined using BCA assay kit (Beyotime Biotechnology, Jiangsu, China) and equalized by adding HEPES-lysis buffer. The samples were subjected to SDS-polyacrylamide gel electrophoresis and the proteins were electrophoretically transferred to Immobilon-P transfer membranes (Millipore, Bedford, MA, USA). The membranes were dipped in blocking buffer [5% bovine serum albumin (BSA) in TBST (Tris-buffered saline plus 0.05% Tween-20, pH 7.4)] overnight at 4 °C. The next day, the membranes were incubated with anti-GluR2 antibody (1:1000, Abcam, Cambridge, UK; catalog no. ab52932), anti-GluR1 antibody (1:1000; Abcam, Cambridge, UK; catalog no. ab109450), anti-PSD-95 antibody (1:1000; Santa Cruz Biotechnology, Santa Cruz, CA, USA; catalog no. sc-71933), anti-PKCζ antibody (1:1000, Santa Cruz Biotechnology, Santa Cruz, CA, USA; catalog no. sc-216), anti-PKCα antibody (1:1000, Santa Cruz Biotechnology, Santa Cruz, CA, USA; catalog no. sc-208), anti-PKCβ antibody (1:1000, Santa Cruz Biotechnology, Santa Cruz, CA, USA; catalog no.sc-209), anti-PKCλ antibody (1:1000, Santa Cruz Biotechnology, Santa Cruz, CA, USA; catalog no. sc-11399), anti-PKCθ antibody (1:1000, Santa Cruz Biotechnology, Santa Cruz, CA, USA; catalog no. sc-212), anti-Na, K-ATPase antibody (1:1000; Abcam, catalog no. ab7671) or anti-β-actin antibody (1:1000, Santa Cruz Biotechnology, Santa Cruz, CA, USA; catalog no. sc-47778) in blocking buffer for 1 h at room temperature on an orbital shaker. After three 5-min washes in TBST buffer, the blots were incubated for 45 min with horseradish peroxidase-conjugated secondary antibody (goat anti-mouse IgG for β-actin and goat anti-rabbit IgG for the others; 1:5000; Santa Cruz Biotechnology, Santa Cruz, CA, USA) diluted in blocking buffer at room temperature on a shaker. The blots were then washed three times for 5 min each in TBST and visualized using the EZ-ECL chemiluminescence detection kit. The band intensities for proteins were quantified by two observers who were blind to the experimental groups using Quantity One 4.4.0 software (Bio-Rad, Hercules, CA, USA). Levels of membrane proteins including GluR1, GluR2 and PSD-95 were normalized to Na, K-ATPase and levels of cytosolic proteins like PKMζ, PKCα, PKCβ, PKCλ and PKCθ were normalized to β-actin^60^. The protein levels in different manipulation groups are presented as fold changes relative to the naive group.

### Immunohistochemistry

The procedure for immunofluorescence was based on our previous study^61^. Seven days after lentivirus microinjection, the rats were anesthetized and perfused with 0.01 M PBS and 4% paraformaldehyde, pH 7.4. The brains were then extracted and put in 4% paraformaldehyde for 24 h. Subsequently, the brains were placed in 30% sucrose for 24–48 h, frozen, and coronally sectioned at 20 μm using a sliding microtome. Brain sections were incubated overnight on a shaker at 4 °C with primary antibody against NeuN (1:2000; Abcam, Cambridge, UK, catalog no. ab177487), followed by incubation with Alexa Fluor goat anti-rabbit 594 (1:500; Life Technologies, Carlsbad, CA, USA, catalog no. A-11037) for 1 h at room temperature. Finally, the brain slices were counterstained with DAPI. Images were captured using an Olympus BX53 fluorescent microscope (Olympus Corporation, Tokyo, Japan).

### Real-time reverse transcription quantitative PCR assay

The procedure for the mRNA assay was modified from a previous study^62^. Total RNA was extracted from tissue punches of the PrL and IL using an RNA Micro kit (Invitrogen). mRNA was reverse-transcribed into cDNA using the First Strand cDNA Synthesis kit (Invitrogen). For the amplification of *PKMζ* cDNA, the sequences of specific primers were the following: forward (5′-CCTTCTATTAGATGCCTGCTCTCC-3′) and reverse (5′-TGAAGGAAGGTCTACACCATCGTTC-3′)^18^. Complementary DNA amplification was performed in a CFX96 real-time PCR system (Bio-Rad, Hercules, CA, USA). The samples were normalized to β-tubulin-4, and the comparative cycle threshold method was used to calculate differences in gene expression between samples^63^. Message RNA status in different manipulation groups is presented as fold changes relative to the naive group.

### DNA methylation assay

The procedure for the DNA methylation assay was based on a previous study^64^. DNA was isolated and purified from tissue punches of the PrL and IL using a DNA Micro kit (Invitrogen). Purified DNA was then bisulfite-modified (BisulFlash DNA modification kit; Epigentek). Quantitative real-time PCR was used to determine the DNA methylation status of the *PKMζ* genes. We focused on the promoter region, and the sequence was provided in previous studies^18^,^65^. Methylation-specific RT-PCR primers were designed using Methprimer software^66^ (available at www.urogene.org/methprimer/). Unmethylated *PKMζ* DNA was amplified using the following primers: forward (5′-ATTTTGGTTTTGTTAGAGTTTGTGT-3′) and reverse (5′-AACCTCTCAATATACTTTCCTCCAAC-3′). Methylated *PKMζ* DNA was amplified using the following primers: forward (5′-ATTTTGGTTTTGTTAGAGTTTGCGT-3′) and reverse (5′-ACCTCTCGATATACTTTCCTCCGAC-3′). RT-PCR amplifications were performed in a CFX96 real-time PCR system (Bio-Rad, Hercules, CA, USA). The samples were normalized to β-tubulin-4, and the comparative cycle threshold method was used to calculate differences in gene expression between samples^14^. DNA methylation status in different manipulation groups is presented as fold changes relative to the naive group.

### Statistical analysis

All of the statistical analyses were performed using SPSS 17.0 software (SPSS, Chicago, IL, USA). Data are expressed as mean ± SEM and were analyzed using analysis of variance (ANOVA) with appropriate between- and within-subject factors for each experiment (see Results). Significant main effects and interactions (*p* < 0.05) in the factorial ANOVAs were further analyzed using Least Significant Difference *post hoc* tests.

## Additional Information

**How to cite this article**: Chen, C. *et al.* Epigenetic modification of PKMζ rescues aging-related cognitive impairment. *Sci. Rep.* **6**, 22096; doi: 10.1038/srep22096 (2016).
